# Rab27a-mediated exosome secretion facilitates classical swine fever virus release and immune evasion

**DOI:** 10.1128/jvi.01488-25

**Published:** 2025-11-11

**Authors:** Ning Li, Yuehan Quan, Bihao Luo, Xinxin Chen, Tao Wang, Zifeng Gong, Liang Zhang, Kangkang Guo

**Affiliations:** 1 College of Veterinary Medicine, Northwest A&F University 12469, Yangling, Shaanxi, China; 2 State Key Laboratory for Animal Disease Control and Prevention, College of Veterinary Medicine, Lanzhou University, Lanzhou Veterinary Research Institute, Chinese Academy of Agricultural Sciences 111658 , Lanzhou, China; University of North Carolina at Chapel Hill, Chapel Hill, North Carolina, USA

**Keywords:** classical swine fever virus (CSFV), exosome, Rab27a, immune evasion, release

## Abstract

Classical swine fever is a highly contagious disease caused by the classical swine fever virus (CSFV), a member of the *Flaviviridae* family. Exosomes are extracellular vesicles that mediate intercellular communication by transferring membrane components and nucleic acids between cells. They play a role in the progression of various infectious diseases and help viruses evade immune responses. However, the impact of CSFV on exosome secretion and whether exosomes facilitate CSFV’s evasion of the host immune response remains unclear. In this study, we first demonstrated that CSFV infection upregulates Rab27a, a small GTPase that regulates exosome secretion by mediating the trafficking and fusion of multivesicular bodies with the plasma membrane. By overexpressing and silencing Rab27a, we assessed its involvement in CSFV replication and release. We also demonstrated an interaction between the CSFV E0 and E2 proteins and Rab27a. Our findings show that Rab27a facilitates the release of exosomes, which, in turn, enhances CSFV spread. These results suggest that CSFV infection upregulates Rab27a, promoting the exosome-mediated release pathway, thereby contributing to viral spread and immune evasion.

**IMPORTANCE:**

Classical swine fever, caused by the classical swine fever virus (CSFV) from the *Flaviviridae* family, is highly contagious. Exosomes, extracellular vesicles that transfer membrane components and nucleic acids, play a role in viral progression and immune evasion. This study shows that CSFV infection upregulates Rab27a, and the CSFV E0 and E2 proteins interact with Rab27a. By manipulating Rab27a expression, we found that Rab27a facilitates exosome release, which enhances CSFV spread. These findings suggest that CSFV exploits Rab27a to promote exosome-mediated release, aiding viral spread and immune evasion.

## INTRODUCTION

Classical swine fever (CSF) is an acute, highly contagious viral disease affecting pigs, caused by the classical swine fever virus (CSFV). This disease poses a significant threat to the global swine industry. Control strategies for CSF typically involve mandatory vaccination combined with the culling of infected animals. However, despite stringent vaccination measures, CSFV continues to re-emerge in multiple outbreaks (1). CSFV is a single-stranded positive-sense RNA virus, and its genome is divided into three major regions: the uncapped 5' untranslated region (containing an internal ribosome entry site), the open reading frame (ORF), and the 3' untranslated region (which lacks a polyA tail) (2). The ORF encodes a polyprotein that is cleaved into four structural proteins (C, Erns, E1, and E2) and eight non-structural proteins (Npro, p7, NS2, NS3, NS4A, NS4B, NS5A, and NS5B) (3).

Rab GTPases are a conserved family of proteins critical for regulating vesicular transport in eukaryotic cells (4). Research has shown that the activation or inhibition of specific Rab GTPases can significantly affect exosome release, including Rab5 and Rab27 (5, 6). Among them, Rab27a plays a pivotal role in regulating the secretion of lysosome-related organelles, such as multivesicular bodies (MVBs) (7). Rab27a undergoes isoprenylation at two conserved cysteine residues at its C-terminus, allowing it to anchor MVBs to the plasma membrane, promoting their docking and fusion with the membrane (8). Inhibition of Rab27a expression impedes MVB docking, leading to a significant reduction in exosome secretion (9).

Various Rab GTPases play crucial roles in the cellular entry of CSFV. For example, CSFV employs the classical clathrin-dependent pathway, which requires Rab5 and Rab7, to infect PK-15 cells (10). Alternatively, the virus can enter porcine alveolar macrophage cells through a caveola-mediated endocytic route, involving Rab5, Rab7, and Rab11 (11). Additionally, Rab14 regulates lipid metabolism and enhances CSFV replication (12). CSFV NS4B also interacts with Rab22a and co-localizes with Rab5 in early endosomes of PK-15 cells, potentially forming a ternary complex that aids in viral entry (13).

Exosomes are small extracellular vesicles (EVs) that facilitate complex intercellular communication, with sizes ranging from 30 to 200 nm (14, 15). Exosomes originate from intraluminal vesicles within MVBs and are transported to the plasma membrane, where they fuse and release exosomes into the extracellular space (16). These vesicles carry intracellular contents, including nucleic acids and proteins, facilitating the intercellular exchange of information and materials (15). Increasing evidence points to a close relationship between viruses and exosomes. For instance, retroviruses use the vesicular budding mechanism to enhance viral budding (17), and hepatitis viruses utilize exosomes for intercellular transmission (18). Additionally, exosomes play a key role in activating innate immunity and antiviral responses, contributing to long-term interactions between viruses and host cells (19–21).

In this study, we investigated how CSFV regulates Rab27a expression and examined its impact on viral replication through overexpression and small interfering RNA (siRNA) knockdown techniques. By focusing on Rab27a’s role in exosome secretion, we aim to uncover how CSFV manipulates host cellular machinery to promote immune evasion and its persistence in the host.

## RESULTS

### CSFV infection enhances Rab27a expression

**Fig 1:**
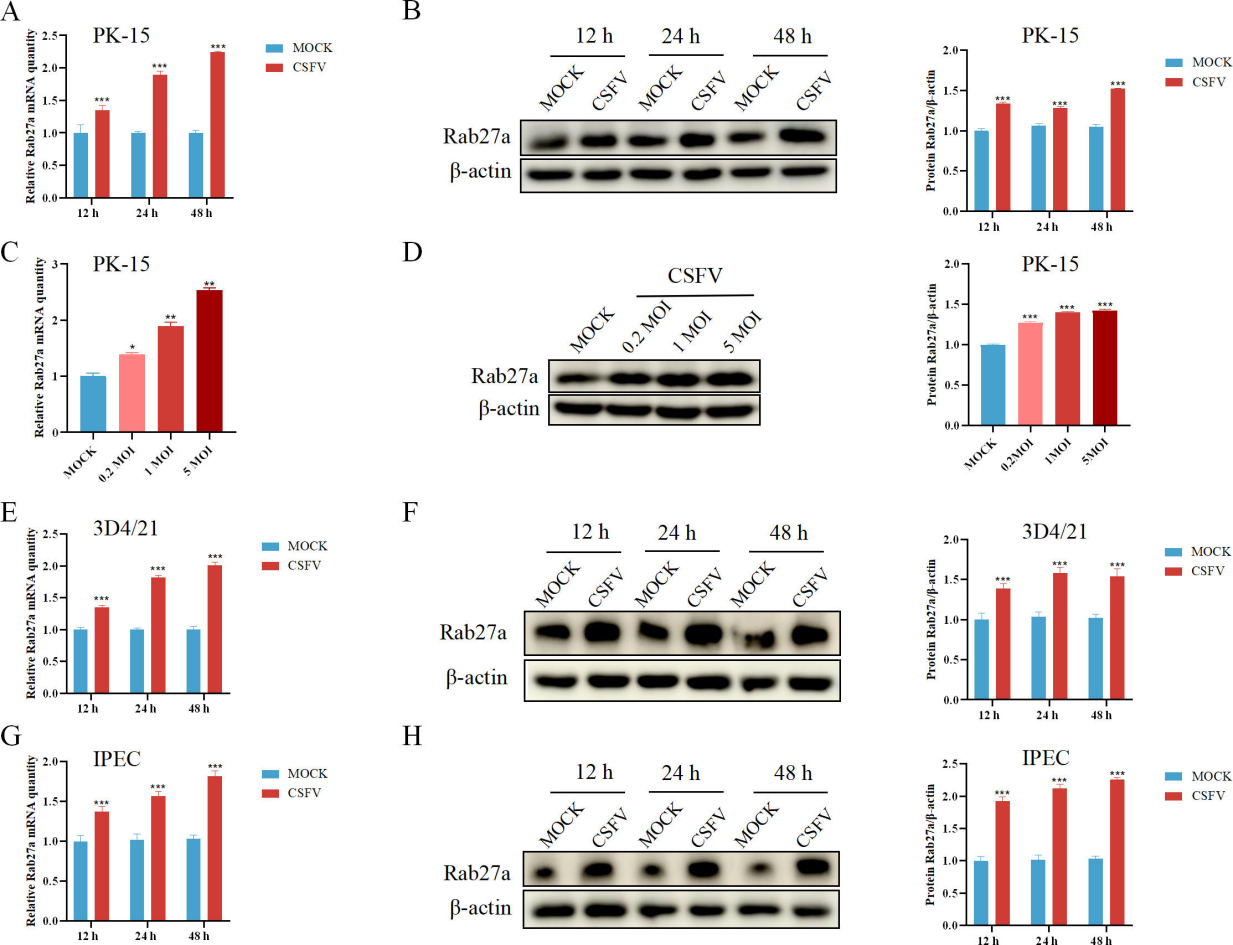
CSFV infection upregulates Rab27a expression. (**A**) PK-15 cells were infected with CSFV (MOI = 1) for 24 and 48 hours, and Rab27a mRNA levels were analyzed by quantitative PCR (qPCR). (**B**) PK-15 cells were infected with CSFV (MOI = 1) for 24 and 48 hours, and Rab27a protein levels were analyzed by western blotting. (**C**) PK-15 cells were infected with CSFV at different MOIs (0, 0.2, 1, and 5) for 48 hours, and Rab27a mRNA levels were analyzed by qPCR. (**D**) PK-15 cells were infected with CSFV at different MOIs (0, 0.2, 1, and 5) for 48 hours, and Rab27a protein levels were analyzed by western blotting. (**E**) 3D4/21 cells were infected with CSFV (MOI = 1) for 24 and 48 hours, and Rab27a mRNA levels were analyzed by qPCR. (**F**) 3D4/2 cells were infected with CSFV (MOI = 1) for 24 and 48 hours, and Rab27a protein levels were analyzed by western blotting. (**G**) IPEC cells were infected with CSFV (MOI = 1) for 24 and 48 hours, and Rab27a mRNA levels were analyzed by qPCR. (**H**) IPEC cells were infected with CSFV (MOI = 1) for 24 and 48 hours, and Rab27a protein levels were analyzed by western blotting. qPCR data were normalized to β-actin mRNA levels, and western blot data were semi-quantified and normalized to β-actin as a loading control. Error bar = SD. *, *P* < 0.05; **, *P* < 0.01; ***, *P* < 0.001. Results shown are representative of three experimental repeats.

To investigate the impact of CSFV infection on Rab27a expression, PK-15 cells were either infected with CSFV at a multiplicity of infection (MOI) of 1.0 or mock-infected for 12, 24, and 48 hours. The relative mRNA and protein expression levels of Rab27a were examined. We found that both mRNA (Fig. 1A) and protein levels (Fig. 1B) of Rab27a were significantly elevated at all indicated time points. We further investigated whether the impact of Rab27a expression is associated with infection MOI. Cells were infected with CSFV at an MOI of 0.2, 1, or a high dose of 5. We observed that both mRNA (Fig. 1C) and protein levels (Fig. 1D) of Rab27a were significantly elevated at all indicated MOI.

To investigate the cellular universality of CSFV infection on Rab27a expression, validation experiments were further conducted in 3D4/21 and IPEC cell lines. Both cell lines were treated with CSFV at an MOI of 1.0 or mock controls for 12, 24, and 48 hours. The relative mRNA and protein expression levels of Rab27a were examined. The results demonstrated that in 3D4/21 cells, both mRNA (Fig. 1E) and protein levels (Fig. 1F) of Rab27a were significantly elevated at all indicated time points. Similarly, in IPEC cells, Rab27a mRNA (Fig. 1G) and protein (Fig. 1H) expression were significantly elevated at all time points.

### Rab27a knockdown inhibited CSFV release

**Fig 2:**
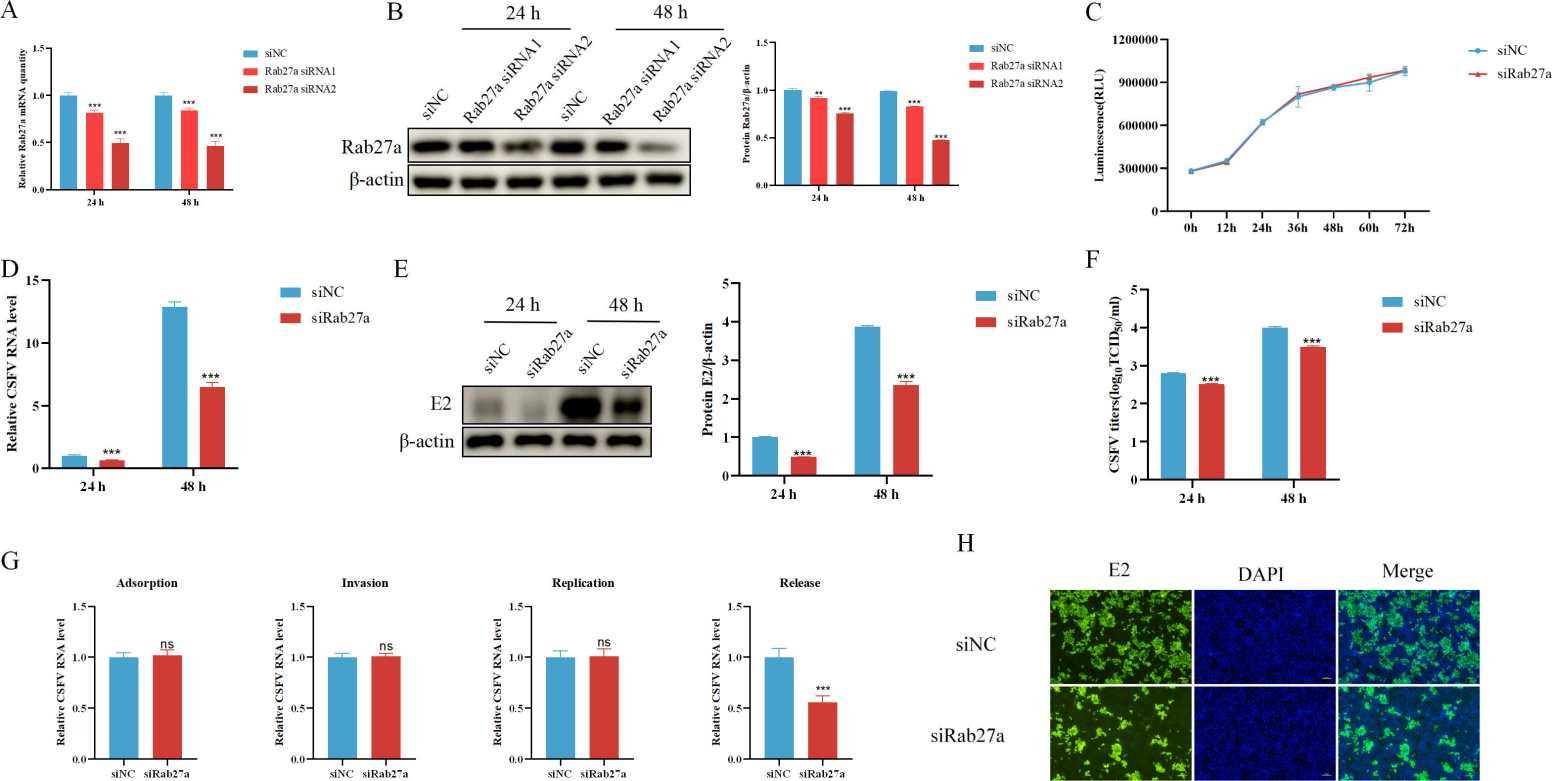
siRNA-mediated knockdown of Rab27a inhibited CSFV release. (**A**) PK-15 cells were transfected with Rab27a siRNA1, siRNA2, or siNC (50 µM) for 24 or 48 hours, and Rab27a mRNA levels were analyzed by qPCR. (**B**) PK-15 cells were transfected with Rab27a siRNA1, siRNA2, or siNC (50 µM) for 24 or 48 hours, and Rab27a protein levels were analyzed by western blotting. (**C**) PK-15 cells were transfected with siRNA2 or siNC (50 µM) for 12, 24, 36, 48, 60, or 72 hours, and cell proliferation was assessed using the CellTiter-Glo assay. (**D and E**) PK-15 cells were transfected with siNC or siRab27a for 24 hours, followed by infection with CSFV (MOI = 1) for 24 or 48 hours. CSFV RNA levels were analyzed by qPCR (**D**), and E2 protein expression was detected by western blotting (**E**). (**F**) CSFV titers in the culture supernatants were determined using the TCID_50_ assay. (**G**) PK-15 cells were transfected with siNC or siRab27a (50 µM) for 24 hours, then incubated with CSFV (MOI = 1) under different conditions: at 4°C for 2 hours to assess viral adsorption, at 37°C for 2 hours to assess viral invasion, at 37°C for 6 hours to assess viral replication, and at 37°C for 9 hours to assess the ratio of viral RNA levels in the culture supernatants to that in the cytoplasm. CSFV RNA levels were analyzed by qPCR. (**H**) Immunofluorescence assays were performed to observe intracellular CSFV propagation in PK-15 cells. qPCR data were normalized to β-actin mRNA levels, and western blot data were semi-quantified and normalized to β-actin as a loading control. Error bar = SD. *, *P* < 0.05; **, *P* < 0.01; ***, *P* < 0.001; “ns” = not significant. Results shown are representative of three experimental repeats.

To investigate the role of Rab27a in CSFV infection, PK-15 cells were transfected with Rab27a-specific siRNA. Knockdown efficiency was assessed using two different siRNA candidates, Rab27a siRNA1 and Rab27a siRNA2, by quantifying Rab27a mRNA and protein levels at 24 and 48 hours post-transfection. The results indicated that both Rab27a siRNA1 and Rab27a siRNA2 efficiently reduced Rab27a expression at both time points and at both the mRNA (Fig. 2A) and protein levels (Fig. 2B) compared to the negative control siRNA (siNC). However, Rab27a siRNA2 exhibited greater knockdown efficiency and was therefore selected for further experiments in this study. To evaluate whether Rab27a knockdown affects cell viability and proliferation, a CellTiter-Glo assay was performed. The results demonstrated no significant changes in cell viability or proliferation following Rab27a knockdown, indicating that the observed effects on CSFV infection were not due to cytotoxicity (Fig. 2C).

Next, we examined the effect of Rab27a knockdown on CSFV proliferation. PK-15 cells were transfected with siRNA for 24 hours, followed by infection or mock infection with CSFV at an MOI of 1.0 for 24 or 48 hours. The relative expression levels of CSFV E2 mRNA and protein were quantified using qPCR and western blot, respectively, while viral titers in the supernatant were measured using the TCID_50_ assay. Compared to control cells transfected with siNC, CSFV proliferation was significantly inhibited in Rab27a-knockdown cells at both time points, at both the mRNA (Fig. 2D) and protein levels (Fig. 2E). Additionally, viral titers in the supernatant were significantly reduced in Rab27a-silenced cells compared to siNC-transfected cells (Fig. 2F). These findings suggest that Rab27a plays a crucial role in CSFV replication.

To further elucidate the role of Rab27a in different stages of the CSFV life cycle, we investigated its effects on viral adsorption, invasion, replication, and release. To assess viral adsorption, cells were incubated with CSFV (MOI 1) at 4°C for 2 hours to allow virus attachment. To examine viral invasion, cells were incubated with CSFV (MOI 1) at 37°C for 2 hours to assess viral entry. To analyze viral replication, cells were incubated with CSFV (MOI 1) at 37°C for 6 hours to evaluate intracellular viral replication. Finally, to assess viral release, cells were incubated with CSFV (MOI 1) at 37°C for 9 hours, and viral levels in the culture supernatant were analyzed by qPCR and indirect immunofluorescence assay (IFA) staining.

The results demonstrated that Rab27a knockdown had minimal impact on viral adsorption, invasion, and replication. However, viral release was significantly inhibited, as evidenced by the decreased detection of CSFV in the supernatant via qPCR (Fig. 2G) and IFA (Fig. 2H). These findings suggest that Rab27a is crucial for efficient CSFV release but does not significantly affect early stages of viral infection.

### Rab27a overexpression promoted CSFV release

**Fig 3:**
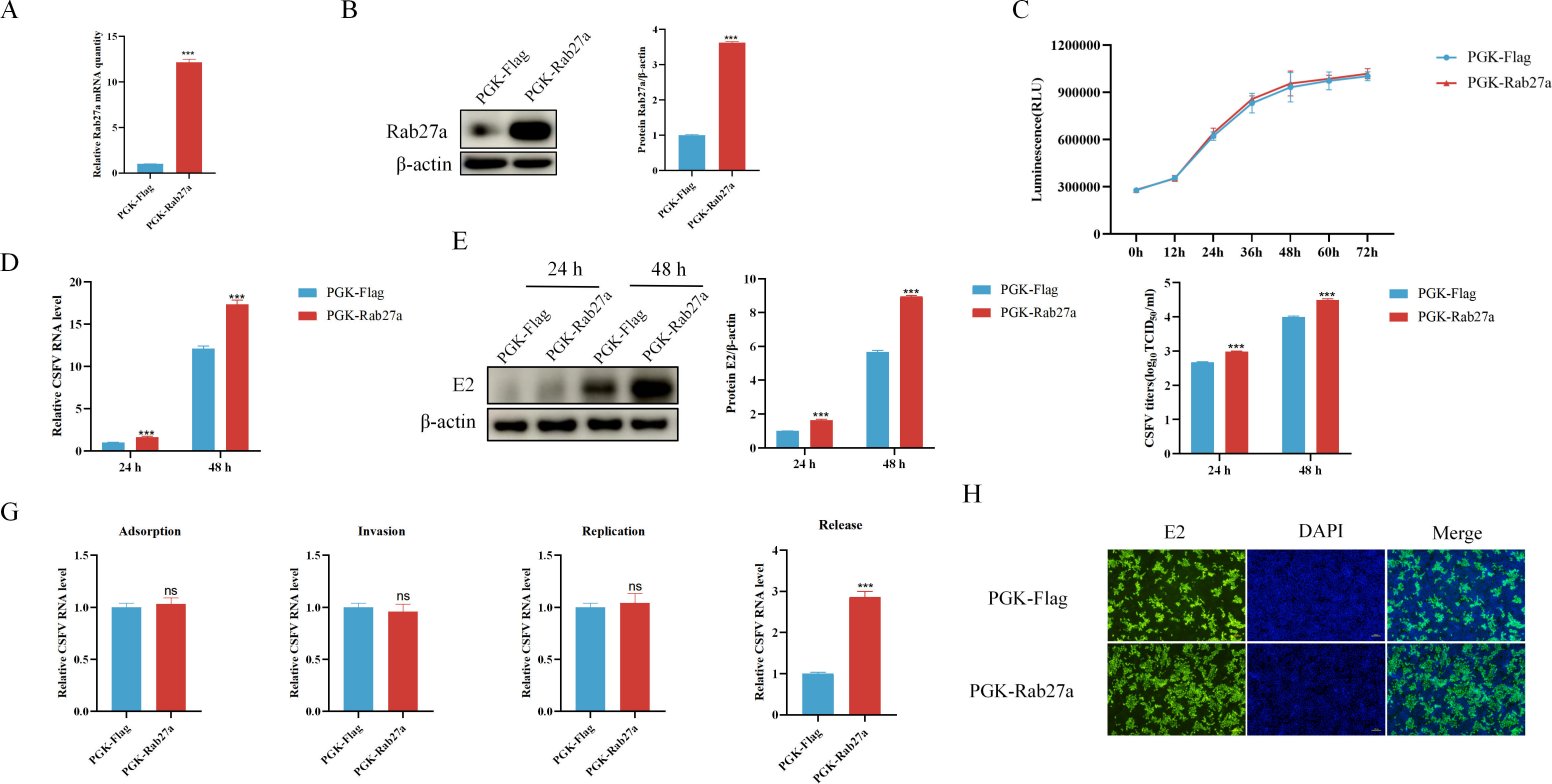
Overexpression of Rab27a enhanced CSFV release. (**A** and **B**) The efficiency of Rab27a overexpression was assessed by qPCR (**A**) and western blotting (**B**). (**C**) Cell proliferation in Rab27a-overexpressing cells was analyzed using the CellTiter-Glo assay. (**D and E**) Cells transfected with PGK-Rab27a or PGK-Flag were incubated with CSFV (MOI = 1) for 24 or 48 hours. CSFV RNA levels were analyzed by qPCR (**D**), and E2 protein expression was detected by western blotting (**E**). (**F**) CSFV titers in the culture supernatants were determined using the TCID_50_ assay. (**G**) Cells transfected with PGK-Rab27a or PGK-Flag were incubated with CSFV (MOI = 1) under different conditions: at 4°C for 2 hours to assess viral adsorption, at 37°C for 2 hours to assess viral invasion, at 37°C for 6 hours to assess viral replication, and at 37°C for 9 hours to assess the ratio of viral RNA levels in the culture supernatants to that in the cytoplasm. CSFV RNA levels were analyzed by qPCR. (**H**) IFAs were performed to observe intracellular CSFV propagation in PK-15 cells. qPCR data were normalized to β-actin mRNA levels, and western blot data were semi-quantified and normalized to β-actin as a loading control. Error bar = SD. *, *P* < 0.05; **, *P* < 0.01; ***, *P* < 0.001; “ns” = not significant. Results shown are representative of three experimental repeats.

To further investigate the role of Rab27a in CSFV infection, we evaluated the effect of Rab27a overexpression on CSFV proliferation. PK-15 cells stably transfected with PGK-Rab27a or the control PGK-Flag plasmid were established. The relative expression of Rab27a was significantly higher at both the mRNA (Fig. 3A) and protein levels (Fig. 3B) in the cell line stably transfected with PGK-Rab27a, compared to the control cell line transfected with PGK-Flag plasmid. Similar to the siRNA knockdown results, overexpression of Rab27a did not result in any significant impact on cell proliferation (Fig. 3C). To assess the impact of Rab27a overexpression on CSFV proliferation, PK-15 cells stably transfected with PGK-Rab27a or the control PGK-Flag plasmid were infected or mock infected with CSFV at an MOI of 1.0 for 24 or 48 hours. The relative expression levels of CSFV E2 mRNA and protein were quantified using qPCR and western blot, respectively, while viral titers in the supernatant were measured using the TCID_50_ assay. Compared to control cells transfected with PGK-Flag plasmid, CSFV proliferation was significantly increased in Rab27a-overexpressing cells at both time points, at both the mRNA (Fig. 3D) and protein levels (Fig. 3E). Additionally, viral titers in the supernatant were significantly higher in Rab27a-overexpressing cells compared to control cells (Fig. 3F).

We also assessed the impact of Rab27a overexpression on different stages of the CSFV life cycle, using the same methods as the siRNA knockdown study. The results demonstrated that Rab27a overexpression had minimal impact on viral adsorption, invasion, and replication. However, viral release was significantly increased, as evidenced by the increased detection of CSFV in the supernatant via qPCR (Fig. 3G) and IFA (Fig. 3H). These findings further corroborate the results from the knockdown study, confirming that Rab27a is crucial for efficient CSFV release but does not significantly affect the early stages of viral infection.

### Interaction of Rab27a with CSFV proteins E0 and E2

**Fig 4:**
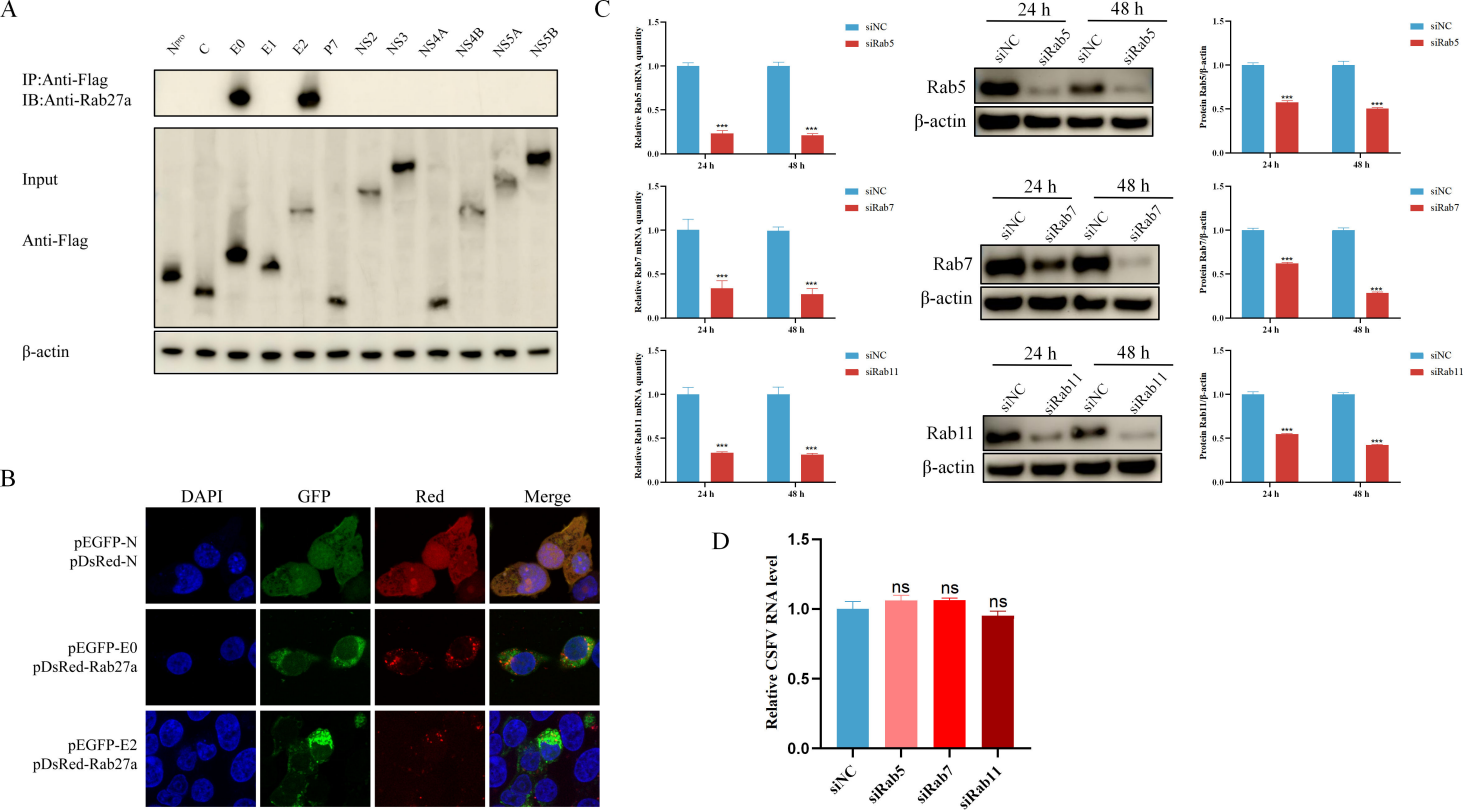
Interaction of Rab27a with CSFV proteins E0 and E2. (**A**) PK-15 cells were transfected with different plasmids of 12 proteins of CSFV for 48 hours. The transfected cells were lysed and immunoprecipitated with anti-Flag followed by western blot analysis using the anti-Flag antibody and anti-Rab27a antibody. (**B**) PK15 cells were transfected with either pEGFP-E0 or pEGFP-E2 in combination with pDsRed-Rab27a. Plasmids pEGFP-N1 and pDsRed-N1 were co-transfected as a control. The transfected cells were fixed with 4% paraformaldehyde and stained with 4',6-diamidino-2-phenylindole (DAPI) (blue) at 48 hours post-transfection. Scale bar = 10 µm. (**C**) PK-15 cells were transfected with specific siRNA (50 µM) targeting Rab5, Rab7, and Rab11 for 24 or 48 hours, and targeted Rab mRNA and proteins levels were analyzed by qPCR and western blotting. (**D**) PK-15 cells were transfected with siNC or specific siRNA (50 µM) targeting Rab5, Rab7, and Rab11 for 24 hours, then incubated with CSFV (MOI = 1) at 37°C for 9 hours to assess the ratio of viral RNA levels in the culture supernatants to those in the cytoplasm. CSFV RNA levels were analyzed by qPCR. qPCR data were normalized to β-actin mRNA levels. Error bar = SD. *, *P* < 0.05; **, *P* < 0.01; ***, *P* < 0.001; “ns” = not significant. Results shown are representative of three experimental repeats.

To investigate the interaction between Rab27a and CSFV proteins, we performed co-immunoprecipitation (Co-IP) experiments. The results showed that Rab27a interacted with the structural proteins E0 and E2 of CSFV, as it was able to co-precipitate with both of these proteins (Fig. 4A). To further validate this interaction, we co-transfected plasmids expressing pEGFP-E0 or pEGFP-E2 with pDsRed1-Rab27a into 293T cells and analyzed the subcellular localization using confocal laser microscopy. The observations revealed that Rab27a significantly co-localized with E0 and E2 in the cytoplasm, providing additional evidence for the interaction between Rab27a and CSFV structural proteins (Fig. 4B).

To assess the specific role of Rab27a during CSFV infection, PK-15 cells were transfected with specific siRNA targeting Rab5, Rab7, and Rab11. Knockdown efficiency was assessed by quantifying targeted Rab mRNA and protein levels at 24 and 48 hours post-transfection. The results confirmed that the siRNAs effectively reduced the expression of the targeted Rab mRNA and proteins (Fig. 4C). Subsequently, we evaluated the impact of silencing these Rab proteins on CSFV release by measuring the ratio of virus titers in the cell supernatant to intracellular virus titers. The results showed that silencing Rab5 and Rab7 slightly increased CSFV release compared to the control group, while silencing Rab11 significantly reduced virus release. However, none of these changes reached statistical significance (Fig. 4D). These results indicate that Rab27A specifically interacts with the CSFV structural proteins E0 and E2 and promotes CSFV release through this interaction, while other Rab family members (Rab5, Rab7, and Rab11) do not significantly affect CSFV release.

### Exosomes were involved in CSFV proliferation

Rab27a, a member of the Rab protein family, is a GTPase associated with the exosome membrane, and it regulates the docking and fusion of the exosome membrane with the plasma membrane, thereby influencing exosome release (16). Evidence suggests that exosomes can facilitate virus transmission between cells (22, 23), and Rab27a may regulate CSFV proliferation through the exosome pathway. To explore whether exosomes are involved in regulating CSFV replication, we examined the impact of exosome inhibition on CSFV replication by treating cells with the exosome-specific inhibitor GW4869.

**Fig 5:**
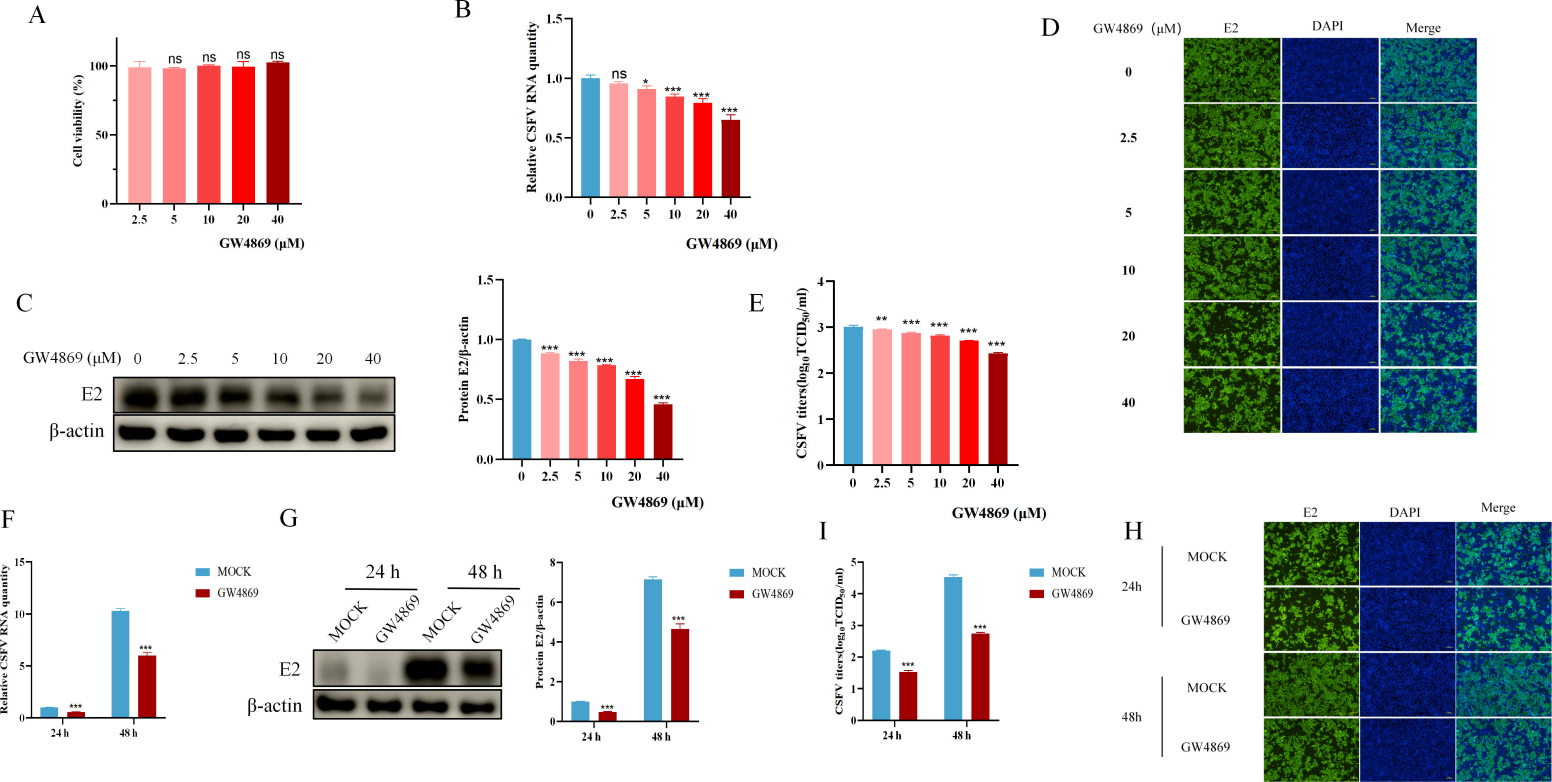
Inhibition of exosome release by GW4869 reduces CSFV propagation. (**A**) PK-15 cells were treated with different concentrations of GW4869 (0, 2.5, 5, 10, 20, and 40 µM), and cell viability was assessed using the CCK-8 assay. (**B and C**) PK-15 cells were treated with different concentrations of GW4869 for 24 hours, followed by infection with CSFV (MOI = 1) for 48 hours. CSFV RNA levels were analyzed by qPCR (**B**), and E2 protein expression was detected by western blotting (**C**). (**D**) IFAs were performed to observe intracellular propagation of CSFV. (**E**) CSFV titers in the culture supernatants were determined using the TCID_50_ assay. (**F and G**) PK-15 cells were treated with GW4869 for 24 hours, followed by infection with CSFV (MOI = 1) for 24 or 48 hours. CSFV RNA levels were analyzed by qPCR (**F**), and E2 protein expression was detected by western blotting (**G**). (**H**) IFAs were performed to observe intracellular propagation of CSFV PK-15 cells. (**I**) CSFV titers in the culture supernatants were determined using the TCID₅₀ assay. qPCR data were normalized to β-actin mRNA levels, and western blot data were semi-quantified and normalized to β-actin as a loading control. Error bar = SD. *, *P* < 0.05; **, *P* < 0.01; ***, *P* < 0.001; “ns” = not significant. Results shown are representative of three experimental repeats.

To determine the optimal concentration of GW4869, we first assessed its cytotoxicity on PK-15 cells across concentrations ranging from 2.5 to 40 µM. The results showed no significant cytotoxic effects on PK-15 cell viability within this concentration range (Fig. 5A). We then investigated the effect of different GW4869 concentrations on CSFV proliferation. PK-15 cells were treated with varying concentrations of GW4869 or mock treated as a control for 24 hours, followed by CSFV infection (MOI = 1) for 48 hours. The results indicated that GW4869 inhibited CSFV proliferation in a dose-dependent manner (Fig. 5B through E). Using 40 µM GW4869, we assessed its impact on CSFV proliferation at different time points post-infection. The results showed that GW4869 effectively inhibited CSFV proliferation at both 24 and 48 hours post-infection (Fig. 4F through I). These findings suggest that exosomes play a crucial role in CSFV proliferation.

### CSFV infection promoted exosome release

**Fig 6:**
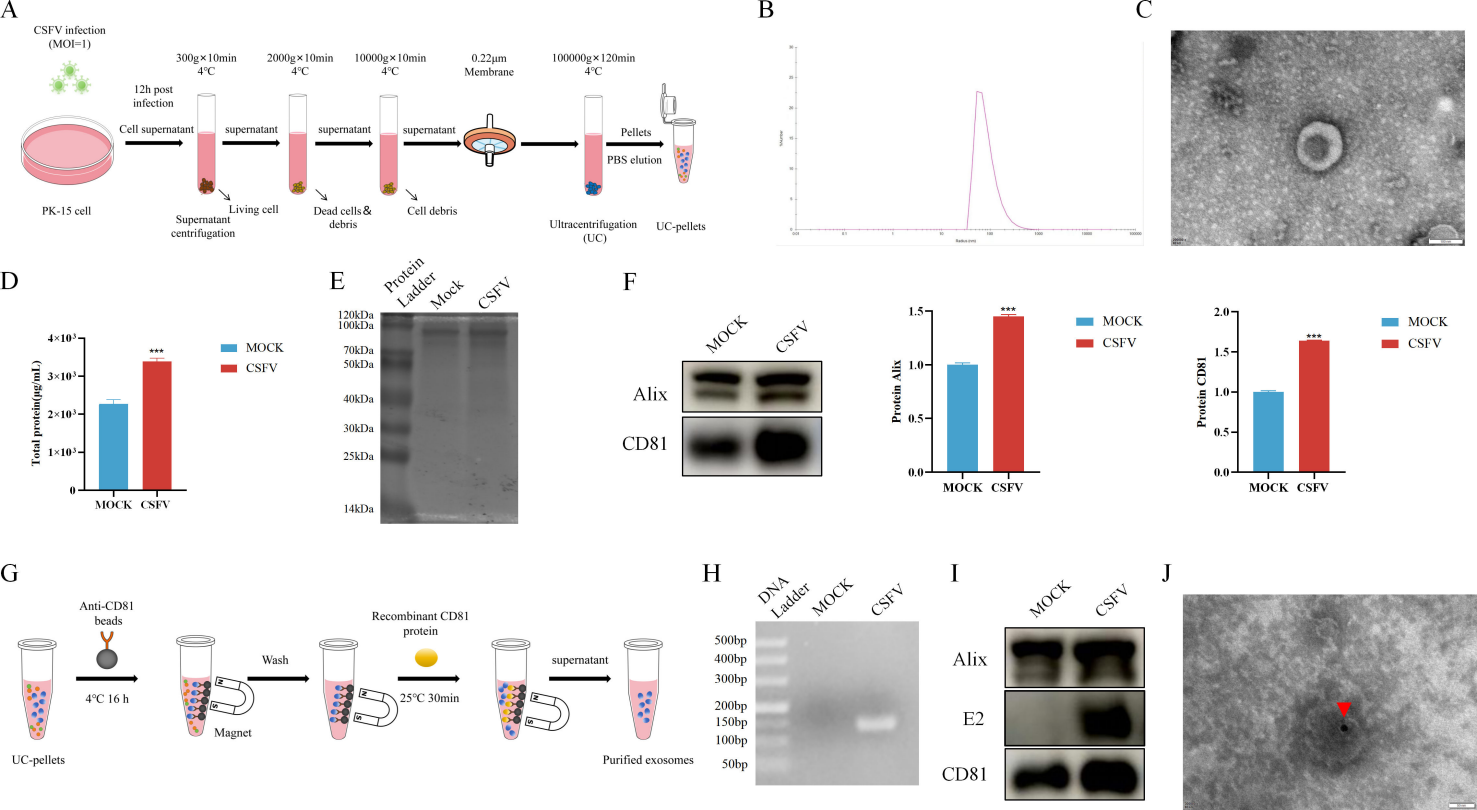
CSFV infection promoted exosome secretion. (**A**) Schematic diagram of exosome isolation. (**B**) NTA was performed to determine the particle size distribution of exosomes isolated from the culture supernatant of CSFV-infected PK-15 cells. (**C**) The morphology of exosomes was visualized using electron microscopy after staining with phosphotungstic acid. (**D**) The total protein concentration of the isolated exosomes was measured using the bicinchoninic acid (BCA) protein assay. (**E**) Exosome proteins were analyzed by SDS-PAGE and stained with Coomassie Brilliant Blue. (**F**) Western blot analysis was performed to assess the expression of the exosome marker proteins Alix and CD81. (**G**) Schematic diagram of exosome purification. (**H**) CSFV RNA in the purified exosomes was detected by PCR. (**I**) CSFV E2 protein in the purified exosomes was detected by western blot analysis. (**J**) Immunoelectron microscopy confirmed the presence of CSFV E2 protein in the purified exosomes.

To investigate whether CSFV affects exosome release, we first examined changes in exosome quantity following CSFV infection. PK-15 cells were inoculated with CSFV, and the cell culture supernatants were collected. Exosomes were isolated from the supernatant by ultracentrifugation, as outlined in the protocol in Fig. 6A, resulting in an ultracentrifugation pellet (UC pellet). Nanoparticle tracking analysis (NTA) revealed that the size of the crude exosome preparation ranged from approximately 30–150 nm (Fig. 6B). Negative staining with 2% phosphotungstic acid followed by transmission electron microscopy (TEM) revealed cup-shaped lipid bilayer vesicles characteristic of exosomes (Fig. 6C), confirming that uniformly sized and stable exosomes were successfully extracted.

To assess changes in exosome release following CSFV infection, we measured the total protein concentration and content of exosomes using a BCA protein assay and Coomassie Brilliant Blue staining. The results indicated that, compared to the control group, the amount of exosomes obtained from the supernatant of CSFV-infected PK-15 cells was significantly increased (Fig. 6D and E). Western blotting was then performed to quantify the exosomal markers CD81 and Alix, and these results were consistent with the protein quantity data (Fig. 6F). These findings suggest that CSFV promotes exosome secretion in PK-15 cells.

To explore whether CSFV is encapsulated within exosomes, we purified the crude exosomes using immunomagnetic beads (Fig. 6G) and analyzed the exosomal nucleic acids by PCR. CSFV nucleic acid fragments were detected in the exosomes derived from infected cells (Fig. 6H). Western blotting also revealed the presence of the E2 protein in the exosomes (Fig. 6I). Furthermore, immunoelectron microscopy showed the presence of CSFV within the exosomes (Fig. 6J).

### Rab27a enhanced CSFV replication by promoting exosome secretion

**Fig 7:**
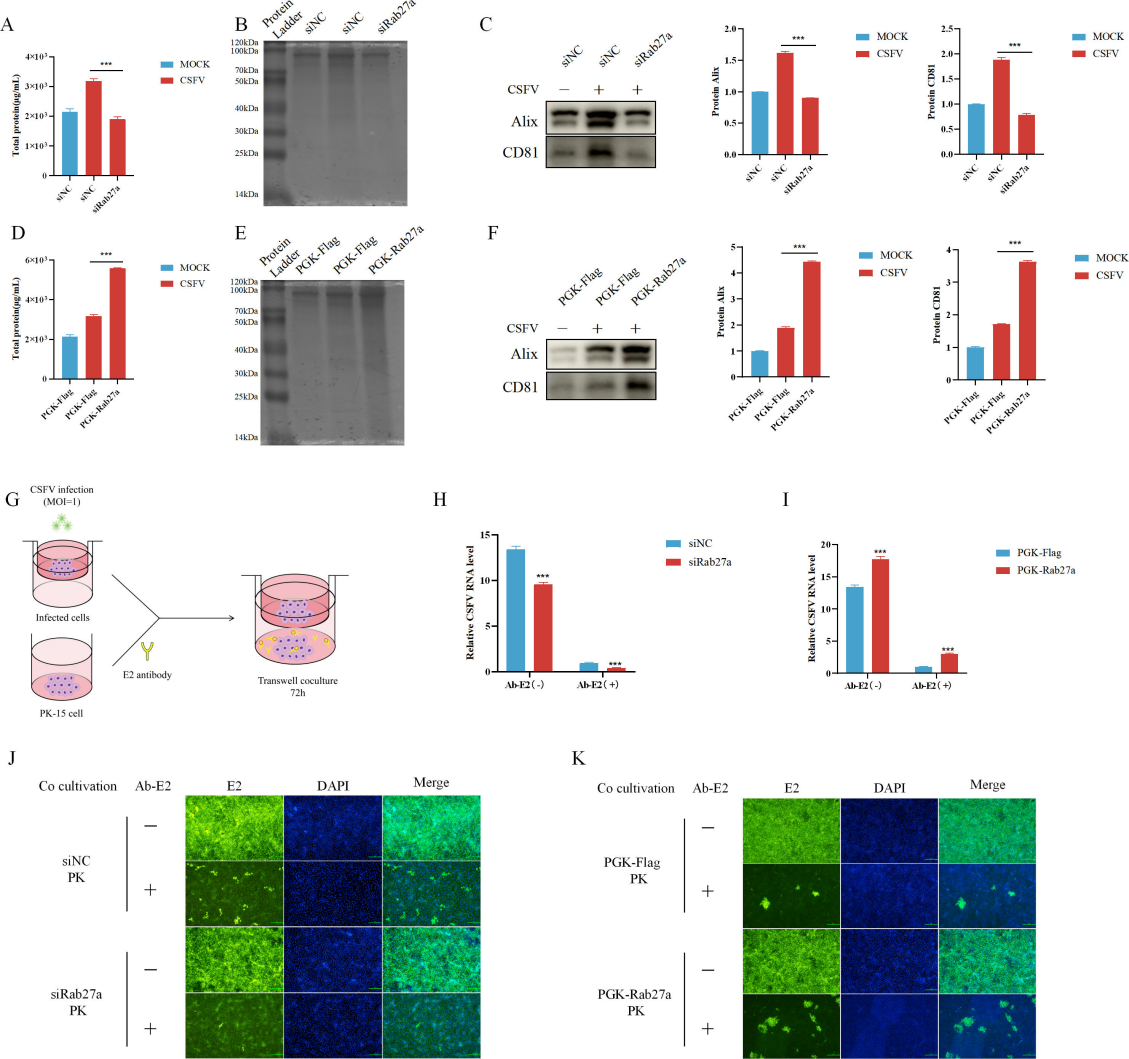
Rab27a enhanced CSFV replication by promoting exosome secretion. (**A–C**) PK-15 cells were transfected with siNC or siRab27a for 24 hours and then infected with CSFV (MOI = 1) for 48 hours. Exosomes were isolated, and their total protein content was quantified using the BCA assay (**A**) and Coomassie Brilliant Blue staining (**B**). Western blot analysis was performed to assess the expression of exosome marker proteins CD81, Alix, and CSFV E2 protein (**C**). (**D–F**) Cells transfected with PGK-Rab27a or PGK-Flag were infected with CSFV (MOI = 1) for 48 hours. Exosomes were isolated, and their total protein content was quantified using the BCA assay (**D**) and Coomassie Brilliant Blue staining (**E**). Western blot analysis was performed to assess the expression of exosome marker proteins CD81, Alix, and CSFV E2 protein (**F**). (**G**) Schematic representation of the Transwell co-culture experiment. PK-15 cells were infected with CSFV (MOI = 1) and co-cultured with uninfected PK-15 cells in the lower chamber for 72 hours, with or without the addition of an E2 neutralizing antibody. (**H and I**) CSFV RNA levels in lower chamber PK-15 cells were measured by qPCR in the Rab27a knockdown group (**H**) and Rab27a overexpression group (**I**). (**J and K**) IFA was used to detect CSFV infection plaques in lower chamber PK-15 cells in the Rab27a knockdown group (**J**) and Rab27a overexpression group (**K**). qPCR data were normalized to β-actin mRNA levels. Error bar = SD. *, *P* < 0.05; **, *P* < 0.01; ***, *P* < 0.001. Results shown are representative of three experimental repeats.

To investigate whether Rab27a influences CSFV release via the regulation of exosome secretion, we first assessed the impact of Rab27a interference on exosome release following CSFV infection. The results showed that, after Rab27a interference, the total protein content of exosomes in CSFV-infected cells was significantly reduced (Fig. 7A and B), and the levels of exosomal marker proteins Alix and CD81 were also decreased (Fig. 7C), in line with the total protein results.

Next, we examined the effect of Rab27a overexpression on exosome release following CSFV infection. The results indicated that Rab27a overexpression led to an increase in the total protein content of exosomes in CSFV-infected cells (Fig. 7D and E), and the levels of exosomal marker proteins Alix and CD81 were elevated (Fig. 7F). These findings suggest that Rab27a enhances CSFV release via the exosome pathway.

Neutralizing antibodies at a concentration of 10× ND_50_ can render 1 MOI of CSFV particles non-infectious, thus preventing free virus particles from infecting cells. In contrast, exosome-mediated viral transmission does not require direct cell-to-cell contact (24). To further investigate whether CSFV release is promoted through the Rab27a-mediated exosome pathway following infection, we designed a Transwell experiment as shown in Fig. 7G. In this experiment, the upper chamber contained PK-15 cells with Rab27a overexpression, Rab27a knockdown (via siRNA), or control groups (either overexpression or siRNA control), all infected with CSFV (MOI = 1.0) for 48 hours. The lower chamber contained PK-15 cells, with a polycarbonate membrane (0.4 µm pores) separating the upper and lower chambers, creating a co-culture system. Excess E2 CSFV neutralizing antibody (10× ND_50_) was added to the lower chamber to neutralize viral particles and prevent free virus particles from infecting the cells. CSFV infection levels in PK-15 cells from both chambers were assessed by qPCR and IFA. The results showed that, after Rab27a interference, the CSFV content in the lower chamber PK-15 cells was reduced (Fig. 7H and I), whereas Rab27a overexpression increased the CSFV content in the lower chamber PK-15 cells (Fig. 7J and K).

These findings further demonstrate that Rab27a promotes CSFV exosome-dependent release. They also suggest that CSFV infection enhances Rab27a expression, which, in turn, increases exosome release and facilitates CSFV release via the exosome pathway.

## DISCUSSION

Following infection, viruses hijack host cellular mechanisms to complete their replication cycle. Rab GTPases play crucial roles in vesicular transport, and many viruses exploit these proteins for invasion, assembly, and release (25). For example, herpes simplex virus type 1 utilizes Rab27a during morphogenesis and release (26). In our previous study, we found that in addition to free propagation, CSFV can also spread between cells through EVs (24). In this study, we investigated the role of Rab27a in CSFV infection and demonstrated that Rab27a expression is upregulated in a variety of CSFV-infected different cells. Functional analysis revealed that Rab27a overexpression enhanced CSFV release, whereas Rab27a knockdown significantly reduced viral release. These findings suggest that Rab27a plays a role in the CSFV lifecycle, particularly in viral egress.

We found that Rab27a directly interacted with the CSFV structural proteins E0 and E2, as evidenced by co-immunoprecipitation and co-localization analysis. These interactions suggest that Rab27a may promote the efficient packaging of virions into exosomes by promoting the recruitment of CSFV structural proteins into exosome biogenesis or transport mechanisms. To further explore the specific role of Rab27a in CSFV release, we extended our study to other Rab family members involved in vesicle trafficking, such as Rab5, Rab7, and Rab11. The results showed that knockdown of these Rab proteins had little effect on CSFV release, and only Rab27a significantly regulated the release of exosome-associated viruses, highlighting the central role of Rab27a in this process.

Rab27a undergoes isoprenylation at conserved cysteine residues in its C-terminal region, facilitating vesicle docking at the plasma membrane (27). Rab27a is also essential for exosome secretion, as its inhibition markedly reduces exosome release (28). Exosomes originate from MVBs and contain various biomolecules, including proteins, lipids, nucleic acids, and viral components. Increasing evidence suggests that viruses utilize exosome-mediated transmission to evade immune responses. Flaviviruses such as dengue virus, West Nile virus, hepatitis C virus (HCV), and Zika virus exploit exosome pathways for intracellular spread (29–32). Moreover, foot-and-mouth disease virus degrades Rab27a to inhibit exosome secretion and evade immune responses (33). Given these findings, we explored whether Rab27a-mediated exosome pathways contribute to CSFV release. Our results indicate that CSFV infection enhances exosome secretion via Rab27a upregulation, and exosome-associated CSFV particles contribute to viral dissemination.

Importantly, we identified intact CSFV particles within exosomes using immunoelectron microscopy, a phenomenon previously reported in other viruses such as Japanese encephalitis virus (JEV) (34) and hepatitis B virus (35). The presence of CSFV RNA and proteins in exosomes, confirmed by PCR and western blot analysis, further supports the hypothesis that exosomes may facilitate CSFV transmission.

To assess whether exosome-mediated viral transfer allows CSFV to evade neutralizing antibodies, we conducted a Transwell experiment. Excess neutralizing antibodies were added to the lower chamber, where no virus was directly introduced. Despite this, CSFV infection was detected in the lower chamber cells, suggesting that exosome-enclosed virions may bypass antibody-mediated neutralization. Similar mechanisms have been described for HCV, where exosome-mediated transmission partially protects viral RNA and proteins from immune clearance (36). Likewise, JEV-derived exosomes have been shown to evade neutralizing antibodies both *in vitro* and *in vivo* (34), aligning with our findings.

In conclusion, this study demonstrates that Rab27a interacts with E0 and E2 and is involved in CSFV release, and CSFV can be transmitted via exosomes, potentially as a strategy for immune evasion. For the first time, we show that CSFV enhances exosome secretion by upregulating Rab27a, and Rab27a interacts with E0 and E2 proteins, thereby promoting virus transmission through the exosome pathway. These findings provide new insights into the role of exosomes in the pathogenesis of CSFV and highlight Rab27a as a potential target for CSF antiviral strategies.

## MATERIALS AND METHODS

### Cell culture and virus

PK-15 and 3D4/21 cell lines were purchased from the American Type Culture Collection (ATCC), and the IPEC cell line is derived from the Veterinary Public Health Laboratory at Northwest A&F University (Shaanxi, China). PK-15 and IPEC were maintained in Dulbecco’s Modified Eagle Medium (DMEM; Gibco, Grand Island, NY, USA) containing 1% penicillin-streptomycin solution (Sigma-Aldrich, St. Louis, MO, USA) and 10% fetal bovine serum (FBS; Gibco, Grand Island, NY, USA). 3D4/21 was maintained in Roswell Park Memorial Institute 1640 (RPMI-1640; Gibco, Grand Island, NY, USA) containing 1% penicillin-streptomycin solution and 10% inactivated FBS. All cell cultures were incubated at 37°C with 5% CO_2_. The CSFV Shimen strain was purchased from the China Veterinary Drug Inspection Institute (Beijing, China) and propagated in PK-15 cells.

### CSFV infection

All experimental work was conducted under appropriate biosafety level (BSL-3) containment in compliance with institutional and national regulations for handling select agents. Cells were infected with CSFV at the indicated MOI and incubated for 2 hours. The inoculum was then removed and replaced with fresh DMEM or RPMI-1640 containing 2% FBS. Supernatants were collected at 9, 24, and 48 hours post-infection.

For exosome collection, cells were first replenished with DMEM containing 2% FBS at 2 hours post-infection. Subsequently, the medium was replaced with serum-free DMEM and incubated for an additional 24 hours.

### Exosome isolation and purification

Exosomes were isolated by differential centrifugation. The cell culture supernatant was first centrifuged at 300 × *g* for 10 minutes at 4°C to remove live cells, and the supernatant was collected. The supernatant was then centrifuged at 2,000 × *g* for 10 minutes to eliminate dead cells, and the supernatant was again collected. The sample was further centrifuged at 10,000 × *g* for 10 minutes to remove cell debris. The resulting supernatant was filtered through a 0.22 µm filter. Exosomes were finally pelleted by ultracentrifugation at 100,000 × *g* for 120 minutes, and the pellet was resuspended in 2 mL of pre-cooled PBS to obtain the purified exosomes.

Exosome purification was then performed using immunomagnetic beads. Fifty microliter of Protein A/G immunomagnetic beads (MCE, catalog number HY-K0202) was washed three times with PBS and incubated with 500 µL of Anti-CD81 antibody (10 µg/mL; Proteintech, catalog number 66866-1-Ig) at room temperature for 2 hours to allow antibody binding. After washing the beads three times with PBS, they were incubated with 1 mL of the ultracentrifuged exosome suspension on a shaker at 4°C overnight. Following incubation, the beads were washed three times with PBS, and 100 µL of recombinant CD81 protein solution (500 µg/mL; Proteintech, catalog number 65195-1-Ig) was added to elute the exosomes. The beads were incubated at 25°C for 30 minutes, and the exosome eluate was collected after allowing the beads to settle on a magnetic stand for 2 minutes. The protein concentration of the isolated exosomes was determined using Coomassie Brilliant Blue staining and a BCA protein assay kit (TargetMol, catalog number C0050).

### Nanoparticle tracking analysis

Briefly, exosome samples were diluted prior to analysis, and the relative concentration was calculated based on the dilution factor. The samples were analyzed using gain adjustment and manual shutter control at a speed of 15 or 30 ms, with shutter speeds between 280 and 560. Data were analyzed using NTA 3.2 software (Malvern Panalytical Ltd, Malvern, Worcestershire, UK), and evaluation was performed using the NanoStar II instrument (Malvern Panalytical Ltd).

### Transmission electron microscopy

To observe the morphology of isolated exosomes, a drop of the exosome sample was placed on a disposable glove, and a copper grid was floated on the droplet for 2 minutes. Excess water was carefully removed from the edge of the grid using filter paper. The sample was then stained by placing the grid onto a droplet of phosphotungstic acid solution for 90 seconds. After air-drying, the copper grid was examined under a TEM at an accelerating voltage of 80 kV to assess the exosome morphology.

For immunoelectron microscopy (immuno-EM), the copper grid with the sample was floated on a fixative solution for 10 minutes, followed by three washes in PBS. To block non-specific binding, the grid was incubated with 50 g/L BSA blocking solution for 30 minutes. The grid was then incubated with a primary antibody (anti-E2) solution for 60 minutes, followed by three PBS washes. The grid was subsequently incubated with a secondary antibody solution for 60 minutes, washed three times with PBS, and stained with phosphotungstic acid. After air-drying, the grid was visualized under a TEM at 80 kV.

### Western blot

The cells or exosome samples were lysed on ice with radio immunoprecipitation assay (RIPA) buffer (MedChem Express, catalog number HY-K0010) supplemented with protease inhibitors. The lysates were centrifuged, and the resulting supernatants were mixed with protein loading buffer, followed by heating at 100°C for 5 minutes to denature the proteins. Protein separation was performed using SDS-PAGE, and the proteins were subsequently transferred to a polyvinylidene fluoride membrane (Merck Millipore, catalog number ISEQ00010).

The membrane was blocked at room temperature for 2 hours using Tris-buffered saline with Tween (TBST) containing 5% non-fat milk. The membrane was incubated overnight at 4°C with primary antibodies: rabbit anti-Rab27a (1:4,000; Proteintech, catalog number 17817-1-AP), rabbit anti-Alix (1:2,000; Proteintech, catalog number 12422-1-AP), and mouse anti-CD81 (1:1,000; Proteintech, catalog number 66866-1-Ig). Following three washes with phosphate buffered saline (PBS), horseradish peroxidase (HRP)-conjugated secondary antibodies (goat anti-mouse IgG, 1:10,000; Immunoway, catalog number RS0001; goat anti-rabbit IgG, 1:10,000; Immunoway, catalog number RS0002) were applied for 2 hours at room temperature. Protein signals were visualized using an enhanced chemiluminescence (ECL) chemiluminescent detection system (mixing solution A and B in equal volumes), and images were captured using a chemiluminescence imaging system. β-actin was used as a loading control.

### Plasmids and siRNA

RNA was extracted from PK-15 cells using TRIzo reagent (Thermo), and cDNA was synthesized using the RevertAid First Strand cDNA Synthesis Kit (Thermo). The pig Rab27a gene was amplified from the cDNA by PCR and cloned into the Piggybac-pgk-3p16-iRFP670-6-IB vector to obtain PGK-Rab27a. The siRNA duplex used in this study was siRab27a. The primers used in this study are listed in Table 1, and the siRNAs used in this study are listed in Table 2.

**TABLE 1:** Primers used for RT-qPCR^*a*^

Primer	Sequence (5′−3′)
β-actin-F	CAAGGACCTCTACGCCAACAC
β-actin-R	TGGAGGCGCGATGATCTT
CSFV-F	GAGAAGGACAGCAGAACTAAGC
CSFV-R	TTACCGCCCATGCCAATAGG
Rab27a-F	AGTGGTGTACAGAGCCAACG
Rab27a-R	TGTCAAGCTGCGAAACCTCT

^*a*^ RT-qPCR, real-time reverse transcription-PCR.

**TABLE 2:** Primers used for siRNA

Primer	Sequence (5′−3′)
Rab27a siRNA1 F	GCUUCCUCAAUGUCAGAAA
Rab27a siRNA1 R	UUUCUGACAUUGAGGAAGC
Rab27a siRNA2 F	GGAAGACCAGAGAGUAGUA
Rab27a siRNA2 R	UACUACUCUCUGGUCUUCC
Rab5 siRNA F	GGAUACAGCUGGUCAAGAA
Rab5 siRNA R	UUCUUGACCAGCUGUAUCC
Rab7 siRNA F	GGAUGACAGACUAGUCACA
Rab7 siRNA R	UGUGACUAGUCUGUCAUCC
Rab11 siRNA F	GGCAGUUCCUACAGAUGAA
Rab11 siRNA R	UUCAUCUGUAGGAACUGCC

### Real-time PCR analysis

Real-time PCR was performed to quantify the expression of *Rab27a*, *CD81*, *Alix*, and viral genomic copies. Total RNA was extracted from cells or tissues using TRIzol reagent (Thermo, catalog number AM9738) and reverse-transcribed into cDNA using the RevertAid First Strand cDNA Synthesis Kit (Thermo, catalog number K1622). SYBR Green Real-Time PCR Master Mix (Takara, catalog number CN830A) was used for amplification, following the manufacturer’s protocol. The reaction conditions were 95°C for 30 s, followed by 40 cycles of 95°C for 5 s and 60°C for 30 s. Primer sequences are available upon request. Relative RNA expression was normalized to *β-actin* using the comparative Ct method.

For viral entry analysis, PK-15 cells were incubated with CSFV (MOI = 5) at 4°C for 1 hour to allow viral attachment, followed by incubation at 37°C for 2 hours to facilitate viral entry. Real-time PCR was used to measure CSFV adsorption (1 hour), viral entry (2 hours), and replication (8 hours).

### Construction of Rab27a-overexpressing PK-15 cell line

PK-15 cells were co-transfected with the PGK-Rab27a or PGK-Flag plasmid as a control, along with transposase PB, using the jetPRIME transfection reagent (Polyplus, 101000046). After 48 hours, cells were selected in medium containing Blasticidin S HCl (5 mg/mL; Beyotime, ST018) to establish a stable cell line. PGK-Flag vector, expressing the Flag protein, served as a control.

### CSFV titration

PK-15 monolayers in 96-well plates were inoculated with 100 µL of serially diluted CSFV. After 1 PBS wash, cells were fixed with 4% formaldehyde for 20 min, permeabilized with 0.5% Triton X-100, and blocked with 5% BSA. Cells were incubated overnight at 4°C with primary antibody, followed by a fluorophore-conjugated secondary antibody for 1 hour in the dark. Nuclei were stained with DAPI, and fluorescence was detected using a confocal microscope (Nikon). Viral titers were calculated as TCID_50_ per half well.

### Cell viability assay

Cell viability was assessed using the Cell Counting Kit-8 (TargetMol, catalog number C0050). Cells were seeded in 96-well plates and cultured for 12, 24, 36, 48, 60, or 72 hours. After the addition of 10 µL of reagent to the culture medium, the mixture was well mixed and incubated at 37°C for 1 hour. The color of the culture medium turned orange, and the absorbance at 450 nm was measured using a microplate reader (Thermo Fisher Scientific). The cell-free group was used as a blank control. The concentration of GW4869 (MCE, catalog number HY-19363) used in this study was determined based on the results of the cell viability assay.

### Cell proliferation assay

Cell proliferation was assessed using the CellTiter-Glo 3D Cell Viability Assay (Promega, catalog number G9681). Cells were seeded in 96-well plates and cultured for 12, 24, 36, 48, 60, or 72 hours. One hundred microliters of CellTiter-Glo 3D Reagent was added to each well. The plates were then gently shaken for 5 minutes to ensure thorough mixing. The plates were incubated at room temperature for 25 minutes. The chemiluminescence values were measured using a luminescence reader (Thermo Fisher Scientific).

### Transwell co-culture assay

Transwell inserts (polycarbonate membrane, 0.4 µm pore size, 24-well plate; Corning, catalog number 3413) containing PK-15 cells with Rab27a overexpression, Rab27a knockdown (via siRNA), or control groups (overexpression or siRNA control) were infected with CSFV (MOI = 1.0) for 48 hours. Uninfected PK-15 cells were seeded in the bottom wells of a 24-well plate. The Transwell inserts with infected cells were then placed into the corresponding wells to establish the co-culture system. The culture medium was supplemented with a neutralizing antibody (10 × ND_50_) to block viral replication, and the medium was replaced every 24 hours. The system was incubated at 37°C for a total of 72 hours.

### Coomassie brilliant blue staining

After SDS-PAGE electrophoresis, protein gels were transferred to a container, and 50 mL of deionized water was added. The gel was microwaved for 3 minutes, then shaken for 5 minutes. It was stained with 20 mL of Coomassie Brilliant Blue Fast Staining Solution for 10–30 minutes. After staining, the gel was washed with 100 mL of deionized water and destained by shaking and replacing the water every 15 minutes for 30–120 minutes until the protein bands were visible.

### Co-IP experiments

PK-15s transfected with 4 µg of plasmids of 12 proteins of CSFV were harvested at 48 hours with western blot and IP lysis buffer (Beyotime, catalog number P0013). Followed by centrifugation for 30 min at 4°C, a quarter of the supernatant was subjected to input assays. The rest were incubated with anti-Flag antibody (1:7,000; Abways, catalog number AB0008) overnight at 4°C, which had been centrifuged and rinsed with TBS. Followed by washing with TBS and boiling in 5× SDS sample buffer, the protein samples were subjected to a western blot with Rabbit anti-Rab27a monoclonal antibody.

### Confocal microscopy

293T cells were seeded onto cell culture coverslips in 24-well plates and incubated for 12 hours at 37°C in a 5% CO_2_ incubator. The plasmids of pEGFP and pDsRed were co-transfected and cultured for 48 hours. Cells were then washed three times with PBS and fixed with 4% paraformaldehyde at room temperature. After three washes with PBS, cells were incubated with DAPI (Biosharp, catalog number BL105A) at 37°C for 10 min and washed three times with PBS. Finally, the images were captured using a Leica TCS SP8 laser scanning confocal microscope (LSM510 META, Zeiss, Germany).

### Statistical analysis

Statistical analyses were performed using GraphPad Prism 8 (GraphPad Software). A Student’s *t*-test or two-way analysis of variance (ANOVA) was used to analyze differences between groups. *P*-values < 0.05 were considered statistically significant.

## Data Availability

The data that support the findings of this study are available within the article.

## References

[1] Moennig V. 2000. Introduction to classical swine fever: virus, disease and control policy. Vet Microbiol 73:93–102. doi:10.1016/s0378-1135(00)00137-1 10785320

[2] Lefkowitz EJ, Dempsey DM, Hendrickson RC, Orton RJ, Siddell SG, Smith DB. 2018. Virus taxonomy: the database of the International Committee on Taxonomy of Viruses (ICTV). Nucleic Acids Res 46:D708–D717. doi:10.1093/nar/gkx932 29040670 PMC5753373

[3] Thiel HJ, Stark R, Weiland E, Rümenapf T, Meyers G. 1991. Hog cholera virus: molecular composition of virions from a pestivirus. J Virol 65:4705–4712. doi:10.1128/JVI.65.9.4705-4712.1991 1870198 PMC248926

[4] Chavrier P, Parton RG, Hauri HP, Simons K, Zerial M. 1990. Localization of low molecular weight GTP binding proteins to exocytic and endocytic compartments. Cell 62:317–329. doi:10.1016/0092-8674(90)90369-p 2115402

[5] Pfeffer SR. 2001. Rab GTPases: specifying and deciphering organelle identity and function. Trends Cell Biol 11:487–491. doi:10.1016/s0962-8924(01)02147-x 11719054

[6] Butler LR, Singh N, Marnin L, Valencia LM, O’Neal AJ, Paz FEC, Shaw DK, Chavez ASO, Pedra JHF. 2024. The role of Rab27 in tick extracellular vesicle biogenesis and pathogen infection. Parasit Vectors 17:57. doi:10.1186/s13071-024-06150-7 38336752 PMC10854084

[7] Goishi K, Mizuno K, Nakanishi H, Sasaki T. 2004. Involvement of Rab27 in antigen-induced histamine release from rat basophilic leukemia 2H3 cells. Biochem Biophys Res Commun 324:294–301. doi:10.1016/j.bbrc.2004.09.050 15465017

[8] Gomes AQ, Ali BR, Ramalho JS, Godfrey RF, Barral DC, Hume AN, Seabra MC. 2003. Membrane targeting of Rab GTPases is influenced by the prenylation motif. Mol Biol Cell 14:1882–1899. doi:10.1091/mbc.e02-10-0639 12802062 PMC165084

[9] Bobrie A, Colombo M, Krumeich S, Raposo G, Théry C. 2012. Diverse subpopulations of vesicles secreted by different intracellular mechanisms are present in exosome preparations obtained by differential ultracentrifugation. J Extracell Vesicles 1. doi:10.3402/jev.v1i0.18397 PMC3760636 24009879

[10] Shi BJ, Liu CC, Zhou J, Wang SQ, Gao ZC, Zhang XM, Zhou B, Chen PY. 2016. Entry of classical swine fever virus into PK-15 cells via a pH-, dynamin-, and cholesterol-dependent, clathrin-mediated endocytic pathway that requires Rab5 and Rab7. J Virol 90:9194–9208. doi:10.1128/JVI.00688-16 27489278 PMC5044825

[11] Zhang YN, Liu YY, Xiao FC, Liu CC, Liang XD, Chen J, Zhou J, Baloch AS, Kan L, Zhou B, Qiu HJ. 2018. Rab5, Rab7, and Rab11 are required for caveola-dependent endocytosis of classical swine fever virus in porcine alveolar macrophages. J Virol 92:e00797-18. doi:10.1128/JVI.00797-18 29769350 PMC6052321

[12] Liu YY, Bai JS, Liu CC, Zhou JF, Chen J, Cheng Y, Zhou B. 2023. The small GTPase Rab14 regulates the trafficking of ceramide from endoplasmic reticulum to golgi apparatus and facilitates classical swine fever virus assembly. J Virol 97:e0036423. doi:10.1128/jvi.00364-23 37255314 PMC10231254

[13] Wang T, Liu Y, Sun Y, Zhang L, Guo K, Zhang Y. 2022. Rab22a cooperates with Rab5 and NS4B in classical swine fever virus entry process. Vet Microbiol 266:109363. doi:10.1016/j.vetmic.2022.109363 35134740

[14] Simons M, Raposo G. 2009. Exosomes--vesicular carriers for intercellular communication. Curr Opin Cell Biol 21:575–581. doi:10.1016/j.ceb.2009.03.007 19442504

[15] Ju Y, Bai H, Ren L, Zhang L. 2021. The role of exosome and the ESCRT pathway on enveloped virus infection. Int J Mol Sci 22:9060. doi:10.3390/ijms22169060 34445766 PMC8396519

[16] Lin Y, Anderson JD, Rahnama LMA, Gu SV, Knowlton AA. 2020. Exosomes in disease and regeneration: biological functions, diagnostics, and beneficial effects. Am J Physiol Heart Circ Physiol 319:H1162–H1180. doi:10.1152/ajpheart.00075.2020 32986962 PMC7792703

[17] Meng B, Ip NCY, Abbink TEM, Kenyon JC, Lever AML. 2020. ESCRT-II functions by linking to ESCRT-I in human immunodeficiency virus-1 budding. Cell Microbiol 22:e13161. doi:10.1111/cmi.13161 31922351 PMC7187348

[18] Feng Z, Hensley L, McKnight KL, Hu F, Madden V, Ping L, Jeong SH, Walker C, Lanford RE, Lemon SM. 2013. A pathogenic picornavirus acquires an envelope by hijacking cellular membranes. Nature 496:367–371. doi:10.1038/nature12029 23542590 PMC3631468

[19] Hong Y, Truong AD, Vu TH, Lee S, Heo J, Kang S, Lillehoj HS, Hong YH. 2022. Exosomes from H5N1 avian influenza virus-infected chickens regulate antiviral immune responses of chicken immune cells. Dev Comp Immunol 130:104368. doi:10.1016/j.dci.2022.104368 35104460

[20] Gong Y, Kong T, Ren X, Chen J, Lin S, Zhang Y, Li S. 2020. Exosome-mediated apoptosis pathway during WSSV infection in crustacean mud crab. PLoS Pathog 16:e1008366. doi:10.1371/journal.ppat.1008366 32433716 PMC7266354

[21] Kouwaki T, Okamoto M, Tsukamoto H, Fukushima Y, Oshiumi H. 2017. Extracellular vesicles deliver host and virus RNA and regulate innate immune response. Int J Mol Sci 18:666. doi:10.3390/ijms18030666 28335522 PMC5372678

[22] Khabir M, Blanchet M, Angelo L, Loucif H, van Grevenynghe J, Bukong TN, Labonté P. 2024. Exosomes as conduits: facilitating hepatitis B virus-independent hepatitis D virus transmission and propagation in hepatocytes. Viruses 16:825. doi:10.3390/v16060825 38932118 PMC11209184

[23] Martínez-Rojas PP, Monroy-Martínez V, Ruiz-Ordaz BH. 2025. Role of extracellular vesicles in the pathogenesis of mosquito-borne flaviviruses that impact public health. J Biomed Sci 32:4. doi:10.1186/s12929-024-01096-5 39754219 PMC11699717

[24] Wang T, Zhang L, Liang W, Liu S, Deng W, Liu Y, Liu Y, Song M, Guo K, Zhang Y. 2022. Extracellular vesicles originating from autophagy mediate an antibody-resistant spread of classical swine fever virus in cell culture. Autophagy 18:1433–1449. doi:10.1080/15548627.2021.1987673 34740307 PMC9225397

[25] Hervé JC, Bourmeyster N. 2018. Rab GTPases, master controllers of eukaryotic trafficking. Small GTPases 9:1–4. doi:10.1080/21541248.2018.1428853 29368995 PMC5902224

[26] Bello-Morales R, Crespillo AJ, Fraile-Ramos A, Tabarés E, Alcina A, López-Guerrero JA. 2012. Role of the small GTPase Rab27a during herpes simplex virus infection of oligodendrocytic cells. BMC Microbiol 12:265. doi:10.1186/1471-2180-12-265 23164453 PMC3554593

[27] Pereira-Leal JB, Seabra MC. 2000. The mammalian Rab family of small GTPases: definition of family and subfamily sequence motifs suggests a mechanism for functional specificity in the Ras superfamily. J Mol Biol 301:1077–1087. doi:10.1006/jmbi.2000.4010 10966806

[28] Ostrowski M, Carmo NB, Krumeich S, Fanget I, Raposo G, Savina A, Moita CF, Schauer K, Hume AN, Freitas RP, Goud B, Benaroch P, Hacohen N, Fukuda M, Desnos C, Seabra MC, Darchen F, Amigorena S, Moita LF, Thery C. 2010. Rab27a and Rab27b control different steps of the exosome secretion pathway. Nat Cell Biol 12:19–30; doi:10.1038/ncb2000 19966785

[29] Vora A, Zhou W, Londono-Renteria B, Woodson M, Sherman MB, Colpitts TM, Neelakanta G, Sultana H. 2018. Arthropod EVs mediate dengue virus transmission through interaction with a tetraspanin domain containing glycoprotein Tsp29Fb. Proc Natl Acad Sci USA 115:E6604–E6613. doi:10.1073/pnas.1720125115 29946031 PMC6048473

[30] Reyes-Ruiz JM, Osuna-Ramos JF, De Jesús-González LA, Palacios-Rápalo SN, Cordero-Rivera CD, Farfan-Morales CN, Hurtado-Monzón AM, Gallardo-Flores CE, Alcaraz-Estrada SL, Salas-Benito JS, Del Ángel RM. 2020. The regulation of flavivirus infection by hijacking exosome-mediated cell-cell communication: new insights on virus-host interactions. Viruses 12:765. doi:10.3390/v12070765 32708685 PMC7412163

[31] Cosset FL, Dreux M. 2014. HCV transmission by hepatic exosomes establishes a productive infection. J Hepatol 60:674–675. doi:10.1016/j.jhep.2013.10.015 24512825

[32] Martínez-Rojas PP, Monroy-Martínez V, Agredano-Moreno LT, Jiménez-García LF, Ruiz-Ordaz BH. 2024. Zika virus-infected monocyte exosomes mediate cell-to-cell viral transmission. Cells 13:144. doi:10.3390/cells13020144 38247836 PMC10814160

[33] Xu G, Xu S, Shi X, Shen C, Zhang D, Zhang T, Hou J, Zhang K, Zheng H, Liu X. 2020. Foot-and-mouth disease virus degrades Rab27a to suppress the exosome-mediated antiviral immune response. Vet Microbiol 251:108889. doi:10.1016/j.vetmic.2020.108889 33223235

[34] Xiong J, Yang L, Nan X, Zhu S, Yan M, Xiang S, Zhang L, Li Q, Yang C, Wang X, Wei N, Chen H, Si Y, Cao S, Ye J. 2025. Extracellular vesicles promote the infection and pathogenicity of Japanese encephalitis virus. J Extracell Vesicles 14:e70033. doi:10.1002/jev2.70033 39783853 PMC11714208

[35] Wu Q, Glitscher M, Tonnemacher S, Schollmeier A, Raupach J, Zahn T, Eberle R, Krijnse-Locker J, Basic M, Hildt E. 2023. Presence of intact hepatitis B virions in exosomes. Cell Mol Gastroenterol Hepatol 15:237–259. doi:10.1016/j.jcmgh.2022.09.012 36184032 PMC9676402

[36] Gu J, Wu J, Fang D, Qiu Y, Zou X, Jia X, Yin Y, Shen L, Mao L. 2020. Exosomes cloak the virion to transmit Enterovirus 71 non-lytically. Virulence 11:32–38. doi:10.1080/21505594.2019.1705022 31885311 PMC6961726

